# Neural substrates of self‐ and external‐preoccupation: A voxel‐based morphometry study

**DOI:** 10.1002/brb3.1267

**Published:** 2019-04-19

**Authors:** Shigeyuki Ikeda, Hikaru Takeuchi, Yasuyuki Taki, Rui Nouchi, Ryoichi Yokoyama, Seishu Nakagawa, Atsushi Sekiguchi, Kunio Iizuka, Sugiko Hanawa, Tsuyoshi Araki, Carlos Makoto Miyauchi, Kohei Sakaki, Takayuki Nozawa, Susumu Yokota, Daniele Magistro, Ryuta Kawashima

**Affiliations:** ^1^ Department of Ubiquitous Sensing Institute of Development, Aging and Cancer, Tohoku University Sendai Japan; ^2^ Division of Developmental Cognitive Neuroscience Institute of Development, Aging and Cancer, Tohoku University Sendai Japan; ^3^ Division of Medical Neuroimaging Analysis, Department of Community Medical Supports Tohoku Medical Megabank Organization, Tohoku University Sendai Japan; ^4^ Department of Radiology and Nuclear Medicine Institute of Development, Aging and Cancer, Tohoku University Sendai Japan; ^5^ Smart Aging Research Center Tohoku University Sendai Japan; ^6^ Department of Advanced Brain Science Institute of Development, Aging and Cancer, Tohoku University Sendai Japan; ^7^ Department of Functional Brain Imaging Institute of Development, Aging and Cancer, Tohoku University Sendai Japan

**Keywords:** cerebellum, gray matter volume, magnetic resonance imaging, posterior cingulate cortex, precuneus, Preoccupation Scale

## Abstract

**Introduction:**

Self‐ and external‐preoccupation have been linked to psychopathological states. The neural substrates underlying self‐ and external‐preoccupation remain unclear. In the present study, we aim to provide insight into the information‐processing mechanisms associated with self‐ and external‐preoccupation at the structural level.

**Methods:**

To investigate the neural substrates of self‐ and external‐preoccupation, we acquired high‐resolution T1‐weighted structural images and Preoccupation Scale scores from 1,122 young subjects. Associations between regional gray matter volume (rGMV) and Preoccupation Scale subscores for self‐ and external‐preoccupation were estimated using voxel‐based morphometry.

**Results:**

Significant positive associations between self‐preoccupation and rGMV were observed in widespread brain areas such as the bilateral precuneus and posterior cingulate gyri, structures known to be associated with self‐triggered self‐reference during rest. Significant negative associations between external‐preoccupation and rGMV were observed only in the bilateral cerebellum, regions known to be associated with behavioral addiction, sustained attention, and reward system.

**Conclusion:**

Our results reveal distinct neural substrates for self‐ and external‐preoccupation at the structural level.

## 1 INTRODUCTION

The disposition to focus more on the self than on others or one's environment and to maintain self‐focused attention is referred to as self‐preoccupation, a state that can be assessed quantitatively using the Self‐Preoccupation Scale (SPS) (Sakamoto, 1998). Self‐preoccupation is implicated in diverse psychopathological states such as depression (Ingram, 1990). Highly self‐preoccupied individuals tend to attribute causes of negative events to themselves by self‐focused attention after the negative event (Sakamoto, 1998). In addition to the SPS, the disposition to focus on the self can be assessed by the private self‐consciousness scale (Fenigstein, Scheier, & Buss, 1975). While similar to the SPS in that it measures self‐focusing tendency, unlike the SPS the private self‐consciousness scale does not consider the disposition to prolonged self‐focus. According to a previous report, depression is more strongly related to the SPS than to the private self‐consciousness scale (Sakamoto, 1998), suggesting the importance of self‐focusing duration in psychopathology. Furthermore, high SPS score was found to be a vulnerability factor for depression (Sakamoto, 1999).

In contrast to self‐preoccupation, the disposition to maintain external focus on a specific object is referred to as external‐preoccupation, which can be assessed by the External‐Preoccupation Scale (EPS) (Sakamoto, 1998). Highly external‐preoccupied individuals tend to devote their attention to their work and exert extreme effort in achieving their goals. This disposition may lead to “burnout” (Freudenberger, 1974), which is also related to depression.

Despite strong relationships to psychopathological states, the neural substrates underlying self‐ and external‐preoccupation remain unclear. To our knowledge, no studies have investigated potential neural substrates using structural magnetic resonance imaging (MRI). In the present study, we aim to provide insight into the information‐processing mechanisms associated with self‐ and external preoccupationat the structural level.

Self‐preoccupation is thought to be associated with self‐referential mental activity in cortical midline structures, including the medial prefrontal cortex (MPFC), posterior cingulate cortex (PCC), and precuneus. Indeed, explicit self‐referential tasks have been found to activate the MPFC, PCC, and precuneus (D'Argembeau et al., 2005; Gusnard, Akbudak, Shulman, & Raichle, 2001; Huang et al., 2014; Johnson et al., 2002; Kelley et al., 2002; Moran, Macrae, Heatherton, Wyland, & Kelley, 2006; Northoff & Bermpohl, 2004; Northoff et al., 2006; Qin & Northoff, 2011). Self‐reference can also occur independently of individual intention during resting‐state (Gusnard & Raichle, 2001; Huang et al., 2016; Lipsman et al., 2014; Wolff et al., 2018), which is hereinafter referred to as self‐triggered self‐reference. Several previous studies have investigated similarities and differences between brain regions activated by explicit self‐reference and self‐triggered self‐reference during rest using positron emission tomography (D'Argembeau et al., 2005) and functional MRI (fMRI) (Whitfield‐Gabrieli et al., 2011). The fMRI study revealed that explicit self‐reference preferentially engaged dorsal MPFC (dMPFC) while self‐triggered self‐reference during rest preferentially engaged the precuneus and both engaged ventral MPFC (vMPFC) and PCC (Whitfield‐Gabrieli et al., 2011). Because self‐preoccupation is the disposition to focus on the self regardless of individual intention, self‐preoccupation may be associated with the precuneus, vMPFC, and PCC, which are activated by self‐triggered self‐reference. In addition, self‐related processes are driven via PCC and moderated by MPFC (Davey, Pujol, & Harrison, 2016). The functions of PCC and MPFC may be implicated in a specific aspect of self‐preoccupation that maintains attention to the self. Therefore, neural substrates of self‐preoccupation should be observed primarily in the precuneus, vMPFC, and PCC but not in the dMPFC.

External‐preoccupation is assumed to have three distinct attributes: propensity for behavioral addiction, sustained attention, and reward system. People with high external‐preoccupation tend to be enthusiastic about things that interest them and to become absorbed by these things. The behavior exhibited by such people is thought to be analogous to behavioral addiction, such as gambling or internet addiction. A recent neuroimaging study reported that gambling disorder patients showed reduced gray matter volume (GMV) in the left supramarginal gyrus and bilateral posterior cerebellum compared to healthy controls (Takeuchi, Tsurumi et al., 2017). Similarly, subjects with Internet addiction disorder showed GMV reduction in the anterior cingulate cortex, supplementary motor area, cerebellum, insula, and inferior temporal gyrus (Weinstein, Livny, & Weizman, 2017 for review). Because external‐preoccupation represents the disposition to maintain external focus, external‐preoccupation may be associated with the ability to maintain focus on a task (i.e., sustained attention). Whole‐brain functional network strength provided a neuromarker for sustained attention to external stimuli, and nodes with the most connections in the functional networks related to sustained attention were located in the cerebellum, temporal, or occipital cortices (Rosenberg et al., 2016). Finally, people with high external‐preoccupation find it difficult to stop a specific action because they expect a reward. This suggests the existence of a relationship between external‐preoccupation and reward system. The cerebellum, as well as canonical reward‐related areas, has been known to be related to reward processing, and is involved in prediction of future rewards (Tanaka et al., 2004), evaluation of unpredicted rewards (Ramnani, Elliott, Athwal, & Passingham, 2004), and reward‐based learning (Thoma, Bellebaum, Koch, Schwarz, & Daum, 2008). Recently, a region of the cerebellum was reported to be specifically activated by verbal encouragement, suggesting that the cerebellum motivates aspects of motor performance (Belkhiria et al., 2017). Furthermore, cerebellar granule cells have been shown to encode the expectation of reward (Wagner, Kim, Savall, Schnitzer, & Luo, 2017). Taken together, the cerebellum is consistently implicated in all three attributes of external‐preoccupation. Therefore, neural substrates of external‐preoccupation should be observed primarily in the cerebellum.

The present study tests two hypotheses: (a) neural substrates of self‐preoccupation include the precuneus, vMPFC, and PCC but not the dMPFC, and (b) neural substrates of external‐preoccupation include the cerebellum. To test our hypotheses, we investigate associations between regional gray matter volume (rGMV) and individual differences in self‐ and external‐preoccupation among a large sample of young adults (more than one thousand). To assess the associations of rGMV to self‐ and external‐preoccupation, we employ voxel‐based morphometry (VBM). Specifically, we perform whole‐brain multiple regression analysis, a method commonly used in neuroimaging research to identify regions associated with specific behavioral outcomes or metrics such as SPS scores.

## 2 MATERIALS AND METHODS

### 2.1 Subjects

The present study is a part of an ongoing project to investigate associations among brain structure and activity, cognitive function, and aging. Recent work includes studies on VBM (Takeuchi et al., 2017a) and resting‐state activity (Ikeda et al., 2017; Takeuchi, Taki, et al., 2017). Therefore, subjects in this study have received psychological tests and MRI scans aside from those described in the present report.

The present study included 1,122 healthy, right‐handed subjects (644 males and 478 females, age 20.7 ± 1.8 years). All subjects were university, college, or postgraduate students or subjects who had graduated from these institutions within 1 year before the experiment. All had normal vision and no history of neurological or psychiatric illness. Handedness was evaluated using the Edinburgh Handedness Inventory (Oldfield, 1971). In accordance with the Declaration of Helsinki, all subjects gave written informed consent for their participation. The present study was approved by the Ethics committee of Tohoku University.

### 2.2 Image acquisition

Magnetic resonance images were acquired as described in our previous work (Takeuchi et al., 2017b) using a 3‐T Philips Intera Achieva scanner equipped with an eight‐channel head coil. We collected high‐resolution T1‐weighted structural images using a magnetization‐prepared rapid gradient echo sequence and the following settings: 240 × 240 matrix, TR = 6.5 ms, TE = 3 ms, FOV = 240 mm, slices = 162, and slice thickness = 1.0 mm. Pads and Velcro tape were used to limit subjects’ motion during scanning.

### 2.3 Psychological measures

All subjects completed the Preoccupation Scale and Raven's Advanced Progressive Matrices (RAPM). The Preoccupation Scale consists of 19 items, each scored on a 5‐point Likert scale, of which 11 items reflect self‐preoccupation, and the remaining items reflect external‐preoccupation (Sakamoto, 1998). Self‐preoccupation is defined as the tendency to focus more on the self than on others or one's environment and to maintain self‐focused attention. In contrast, external‐preoccupation is defined as the tendency to maintain external focus on a specific object. Each subscale (SPS, EPS) is calculated by summing scores of the items. The RAPM is a task to assess nonverbal reasoning ability (Raven, 1998). Intelligence quotient (e.g., RAPM score) is associated with attention deficit hyperactivity disorder (ADHD) (Rommel, Rijsdijk, Greven, Asherson, & Kuntsi, 2015). The RAPM score was, therefore, used as a nuisance covariate in subsequent second‐level analysis.

To check association between SPS/EPS and personality traits (i.e., neuroticism, extraversion, openness, agreeableness, and conscientiousness), the subjects were asked to complete a 60‐item Japanese version (5‐point scale) of the NEO Five‐Factor Inventory (NEO‐FFI) (Costa & MacCrae, 1992; Shimonaka, Nakazato, Gondo, & Takayama, 1999). Note that one subject did not complete the NEO‐FFI. To establish the association between SPS/EPS and personality traits, we calculated Pearson's correlation coefficients.

### 2.4 Preprocessing of structural data

We employed the same preprocessing procedures as described previously (Takeuchi et al., 2017b). The preprocessing was performed using Statistical Parametric Mapping software (SPM12; Wellcome Department of Cognitive Neurology, London, UK, https://www.fil.ion.ucl.ac.uk/spm/) implemented in Matlab (Mathworks Inc., Natick, MA). Individual T1‐weighted structural images were segmented into six tissues using the new segmentation algorithm in SPM12. We used default parameters for this new segmentation process except for the following: the Thorough Clean option was used for removing any odd voxels, affine regularization was performed with the International Consortium for Brain Mapping template for East Asian brains, and the sampling distance was set at 1 mm. Separate gray matter and white matter tissue probability maps (TPMs) were created in the segmentation process. The diffeomorphic anatomical registration through exponentiated lie algebra (DARTEL) registration process was then performed on these TPMs. We created the template for the DARTEL algorithm using imaging data from 800 subjects (400 males and 400 females). The following procedures were the same as in our previous work (Takeuchi et al., 2015). The created template was then used to perform the DARTEL procedures using default parameters for all subjects. Individual images were then spatially normalized to Montreal Neurological Institute space (1.5 × 1.5 × 1.5 mm^3^ voxels). Furthermore, to determine regional differences in the absolute amount of brain tissue, a volume change correction was performed by modulating each voxel with the Jacobian determinants derived from spatial normalization (Ashburner & Friston, 2000). Individual images were then smoothed by convolution with a Gaussian kernel of 8‐mm full‐width at half‐maximum.

### 2.5 Second‐level analysis

To investigate associations of rGMV with SPS and EPS, a whole‐brain multiple regression analysis was performed using SPM12. Sex, age, RAPM score, and total intracranial volume (TIV) were included as nuisance covariates in the second‐level analysis. Sex and age are commonly used as nuisance covariates in VBM, while RAPM score is reportedly related to ADHD (Rommel et al., 2015) and so was also used as a nuisance covariate. Regressing out TIV permits comparison of regional volume differences unrelated to differences in total brain volume. The SPS and EPS scores were included as simultaneous covariates of interest in a multiple regression model to reveal associations unique to each Preoccupation Scale subscale. Only voxels with values exceeding an absolute threshold of 0.05 were targeted for the analysis. This threshold is widely used in VBM studies (Beal, Gracco, Lafaille, & Nil, 2007; Focke, Thompson, & Duncan, 2008; Mueller et al., 2006; Nauchi & Sakai, 2009; Schaufelberger et al., 2007; Takeuchi et al., 2010, 2011, 2017b; White, Alkire, & Haier, 2003). To test whether the associations between rGMV and the Preoccupation Scale subscores were statistically significant, a permutation‐based voxel‐wise nonparametric test (5,000 permutations) was performed using the threshold‐free cluster enhancement (TFCE) toolbox (http://dbm.neuro.uni-jena.de/tfce/; r95) (Smith & Nichols, 2009). The TFCE method can estimate voxel‐wise values (TFCE values) representing the amount of cluster‐like local spatial support without an arbitrary cluster‐forming threshold as commonly used in the traditional approach. The resulting TFCE maps were thresholded at a family‐wise error (FWE) corrected *p* < 0.05.

In order to check the influence of RAPM and age as nuisance covariates, we performed additional whole‐brain multiple regression analyses using two models (i.e., a model without RAPM and a model without age). Furthermore, we tested whether there was an interaction effect between SPS and EPS on rGMV. In order to check the interaction effect, we performed an additional analysis using the regression model which included the interaction term between SPS and EPS. The procedures of these additional analyses were similar to the previous paragraph mentioned above except for the regression models.

## 3 RESULTS

### 3.1 Behavioral data

Ages, RAPM scores, TIVs, SPS scores, and EPS scores of the total group (both sexes), the male‐only group, and the female‐only group are shown in Table 1. Pearson's correlations were computed to test associations among age, TIV, and behavioral measures (Table S1). There were significant correlations between age and SPS, and between RAPM and SPS. These results suggest the importance of using age and RAPM as nuisance covariates.

**Table 1:** Summary of subject demographics and psychometric results

	All			Male			Female		
	Mean	Range	*SD*	Mean	Range	*SD*	Mean	Range	*SD*
Age	20.7	18–27	1.8	20.8	18–27	1.9	20.5	18–27	1.6
RAPM	28.5	13–36	3.9	28.8	13–36	3.9	28.1	15–36	3.8
TIV [cm^3^]	1,535	1,197–2,018	141	1,612	1,352–2,018	119	1,430	1,197–1,737	93
SPS	33.1	11–55	8.9	33.5	11–55	8.9	32.5	11–55	8.9
EPS	27.0	10–40	5.4	27.3	13–40	5.2	26.6	10–40	5.7

*SD*: standard deviation; RAPM: Raven's Advanced Progressive Matrices; TIV: total intracranial volume; SPS: Self‐Preoccupation Scale; EPS: External‐Preoccupation Scale.

We investigated correlations between SPS, EPS, and five personality traits assessed by the NEO‐FFI (Table S2). SPS showed significant correlations with all the personality traits, and in particular, showed a strong positive correlation with neuroticism (*r* = 0.57). On the other hand, EPS showed significant correlations with some personality traits, and in particular, showed a strong positive correlation with conscientiousness (*r* = 0.29).

### 3.2 Association between SPS and rGMV

**Figure 1:**
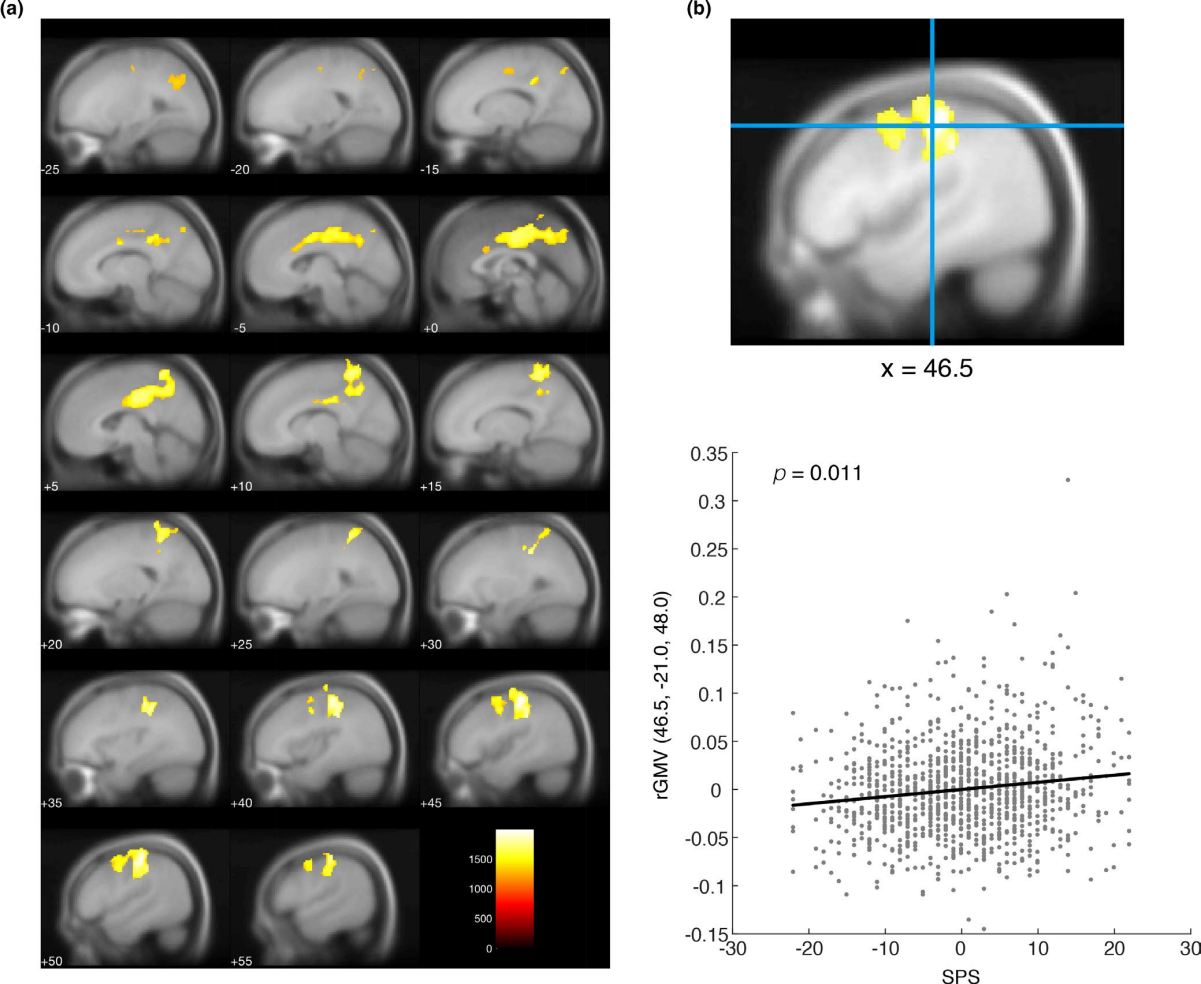
Significant positive associations between Self‐Preoccupation Scale (SPS) and regional gray matter volume (rGMV). (a) Regions demonstrating significant positive associations with SPS scores. Threshold‐free cluster enhancement (TFCE) maps thresholded with a family‐wise error (FWE)‐corrected *p* < 0.05 were overlaid on the avg305T1 template using SPM12. The color bar represents the TFCE magnitude. Warm colors represent positive associations. (b) A scatter plot showing the relationship between SPS scores and rGMV at the peak voxel. The peak voxel was located in the right postcentral gyrus. For the scatter plot, the mean value was subtracted from SPS scores, and the nuisance covariates were regressed out from rGMV at the peak voxel. A black line within the scatter plot shows rGMV predicted from SPS scores

To investigate the potential associations between SPS and rGMV, we performed whole‐brain multiple regression analysis and tested the statistical significance of individual voxels with a voxel‐wise (FWE‐corrected) threshold of *p* < 0.05. Significant positive associations between SPS and rGMV were observed in widespread brain areas including the bilateral precuneus and posterior cingulate gyri (PCG) but not the dMPFC (Figure 1 and Table 2). These results support our first hypothesis. However, contrary to our expectations, no significant associations were observed in the vMPFC. In addition to the precuneus and PCG, significant positive associations with SPS were found in the bilateral superior parietal lobules, bilateral precentral gyrus, bilateral middle cingulate gyrus (MCG), bilateral cuneus, right postcentral gyrus, right supramarginal gyrus, right middle frontal gyrus, left angular gyrus, left supplementary motor cortex, and left superior frontal gyrus. No significant negative associations between SPS and rGMV were observed.

**Table 2:** Brain areas showing positive associations with Self‐Preoccupation Scale

	Anatomical areas (number of significant voxels of each anatomical area)	*x*	*y*	*z*	TFCE	*P*_FWE_	Cluster size
Cluster 1	R precuneus (1,518)	10.5	−48	63	1,766.47	0.019	14,247
	R postcentral gyrus (1,216)	46.5	−21	48	1,987.07	0.011	
	R superior parietal lobule (1,041)	33	−37.5	45	1,869.08	0.014	
	R precentral gyrus (743)	43.5	−16.5	63	1,612.88	0.024	
	R supramarginal gyrus (732)	49.5	−25.5	49.5	1,922.06	0.013	
	R middle cingulate gyrus (725)	4.5	−21	36	1,738.58	0.019	
	R posterior cingulate gyrus (472)	4.5	−28.5	40.5	1,733.41	0.019	
	R middle frontal gyrus (59)	46.5	7.5	52.5	1,505.51	0.029	
	R precentral gyrus medial segment (24)	3	−27	48	1,575.26	0.026	
	R postcentral gyrus medial segment (23)	10.5	−42	63	1,637.5	0.023	
	R cuneus (7)	1.5	−75	36	1,453.51	0.034	
	L middle cingulate gyrus (1,008)	0	−18	39	1,715.65	0.02	
	L precuneus (851)	0	−57	42	1,644.57	0.023	
	L posterior cingulate gyrus (621)	0	−27	45	1,687.56	0.021	
	L superior parietal lobule (344)	−15	−73.5	46.5	1,385.42	0.039	
	L precentral gyrus medial segment (206)	−3	−25.5	46.5	1,652.54	0.023	
	L angular gyrus (103)	−27	−66	34.5	1,349.39	0.043	
	L supplementary motor cortex (10)	−4.5	−18	46.5	1,317.08	0.046	
	L precentral gyrus (6)	−24	−13.5	52.5	1,288.44	0.05	
	L cuneus (1)	0	−76.5	36	1,285.12	0.05	
	L superior frontal gyrus (1)	−22.5	−12	52.5	1,285.12	0.05	
	*R cerebral white matter (2,634)	45	−22.5	49.5	1,983.67	0.011	
	*L cerebral white matter (1,205)	−9	−33	37.5	1,629.66	0.023	
	*Unknown (697)	51	−24	49.5	1,912.4	0.013	
Cluster 2	*R cerebral white matter (3)	13.5	−42	28.5	1,285.12	0.05	3

Note

Labeling of brain areas is conducted using custom Matlab scripts and labels_Neuromorphometrics.nii in SPM12. The coordinates of the peak voxel of each brain area are shown as x, y, and z. Asterisks represent white matter and areas that could not be labeled. The TFCE magnitude and corrected p‐value (FWE) for each peak voxel were shown. Cluster size represents the number of voxels which each cluster includes.

R: right; L: left; FWE: family‐wise error.

### 3.3 Association between EPS and rGMV

**Figure 2:**
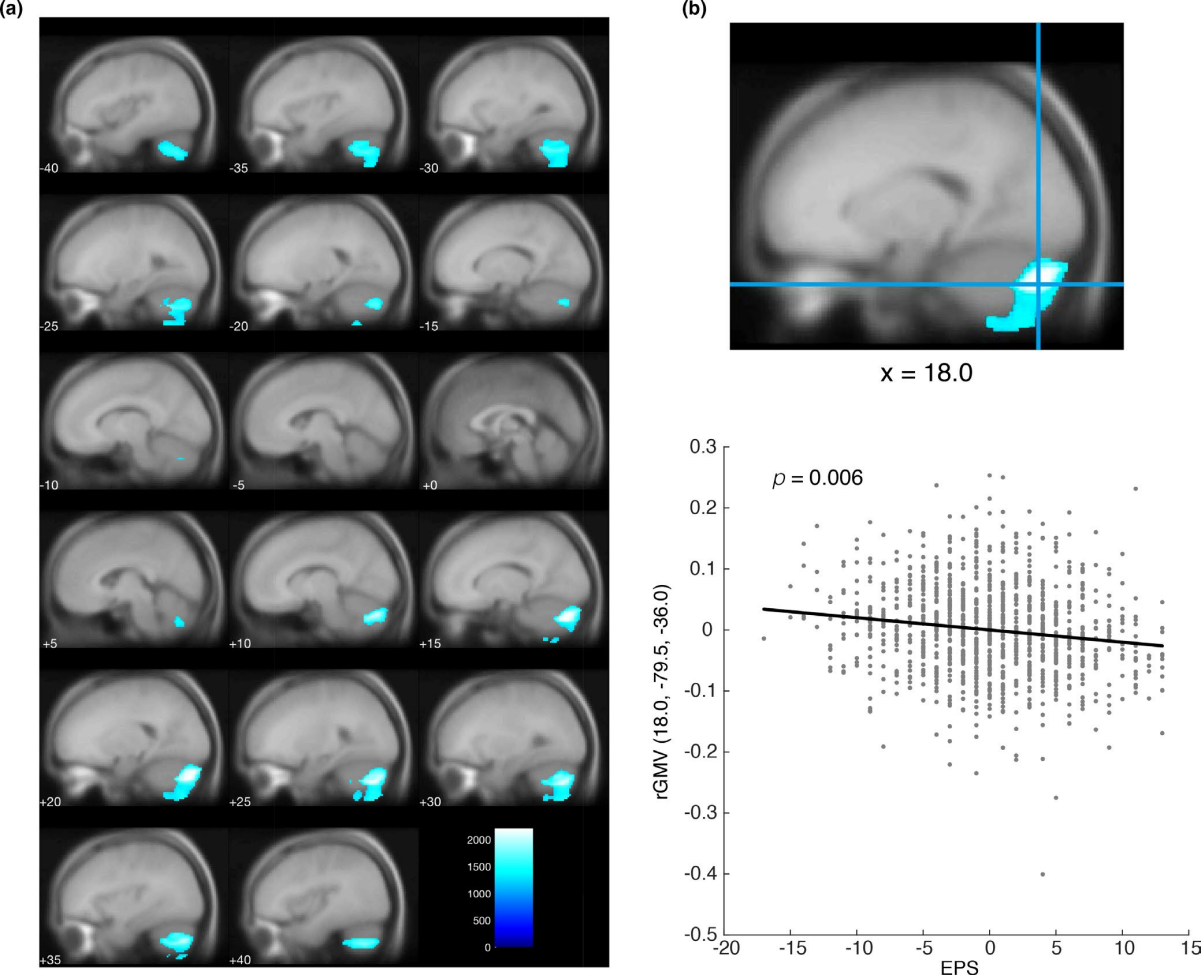
Significant negative associations between External‐Preoccupation Scale (EPS) and regional gray matter volume (rGMV). (a) Regions demonstrating significant negative associations with EPS scores. Threshold‐free cluster enhancement (TFCE) maps thresholded with a family‐wise error (FWE)‐corrected *p* < 0.05 were overlaid on the avg305T1 template using SPM12. The color bar represents the TFCE magnitude. Cool colors represent negative associations. (b) A scatter plot showing the relationship between EPS scores and rGMV at the peak voxel. The peak voxel was located in the right cerebellum exterior. For the scatter plot, the mean value was subtracted from EPS scores, and the nuisance covariates were regressed out from rGMV at the peak voxel. A black line within the scatter plot shows rGMV predicted from EPS scores

To investigate the potential associations between EPS and rGMV, we performed whole‐brain multiple regression analysis and tested the statistical significance of individual voxels with a voxel‐wise threshold (FWE‐corrected) of *p* < 0.05. Significant negative associations were found between EPS and rGMV only in the bilateral cerebellum (Figure 2 and Table 3), supporting our second hypothesis. No significant positive associations between EPS and rGMV were observed.

**Table 3:** Brain areas showing negative associations with External‐Preoccupation Scale

	Anatomical areas (number of significant voxels of each anatomical area)	*x*	*y*	*z*	TFCE	*P*_FWE_	Cluster size
Cluster 1	L cerebellum exterior (3,096)	−21	−76.5	−40.5	1,685.46	0.018	4,468
	*L cerebellum white matter (969)	−27	−55.5	−40.5	1,713.01	0.017	
	*Unknown (403)	−24	−57	−63	1,472.71	0.028	
Cluster 2	R cerebellum exterior (5,778)	18	−79.5	−36	2,204.93	0.006	7,407
	Cerebellar vermal lobules viii–x (91)	7.5	−67.5	−37.5	1,738.94	0.017	
	Cerebellar vermal lobules vi–vii (2)	6	−66	−31.5	1,309.33	0.045	
	*R cerebellum white matter (1,022)	16.5	−75	−37.5	2,166.41	0.007	
	*Unknown (514)	18	−91.5	−27	1,873.52	0.013	

Note

Labeling of brain areas is conducted using custom Matlab scripts and labels_Neuromorphometrics.nii in SPM12. The coordinates of the peak voxel of each brain area are shown as x, y, and z. Asterisks represent white matter and areas that could not be labeled. The TFCE magnitude and corrected p‐value (FWE) for each peak voxel were shown. Cluster size represents the number of voxels which each cluster includes.

R: right; L: left; FWE: family‐wise error.

### 3.4 Influence of RAPM and age, and an interaction effect between SPS and EPS

We performed the additional whole‐brain multiple regression analyses using two regression models (i.e., a model without RAPM and a model without age) to check whether our results were influenced by RAPM and age. When using the model without RAPM, we observed similar results to those observed in the model with RAPM (Table S3 and S4). When using the model without age, we observed brain regions positively associated with SPS (Table S5), and many of the significant brain regions were not observed in the model with age. Moreover, we observed brain regions negatively associated with EPS in the model without age (Table S6), and the significant brain regions overlapped substantially with those observed in the model with age. No other significant associations were observed.

We performed the additional analysis using the regression model which included the interaction term between SPS and EPS to check the interaction effect between SPS and EPS on rGMV. As a result, we observed no brain regions showing significant associations with the interaction term.

## 4 DISCUSSION

The present study investigated associations between rGMV and both SPS and EPS in a large sample of young adults. We found significant voxel‐wise positive associations between SPS and rGMV distributed over the bilateral precuneus and PCG but not the dMPFC, supporting our first hypothesis that self‐preoccupation is supported by brain regions related to self‐triggered self‐reference. However, contrary to our expectation, no significant associations with SPS score were observed in vMPFC. Alternatively, significant positive associations between rGMV and SPS were widely observed in the bilateral superior parietal lobules, bilateral precentral gyrus, bilateral MCG, bilateral cuneus, right postcentral gyrus, right supramarginal gyrus, right middle frontal gyrus, left angular gyrus, left supplementary motor cortex, and left superior frontal gyrus, implicating these regions in self‐preoccupation. We also found significant negative associations between EPS and rGMV only in the cerebellum, supporting our second hypothesis that external‐preoccupation is subserved by regions implicated in behavioral addiction, sustained attention, and reward system. Collectively, our study reveals distinct neural substrates for self‐ and external‐preoccupation at the structural level.

The associations of SPS with the precuneus and PCG suggest that self‐preoccupation occurs independently of individual intention during resting‐state because activity of the precuneus and PCG is related to self‐triggered self‐reference (Whitfield‐Gabrieli et al., 2011). On the other hand, distinct functions of the precuneus and PCG in different aspects of self‐preoccupation are suggested by the following previous findings. First, activation in PCG was associated with incidence of task‐unrelated thought, while no significant associations were observed in the precuneus (Mckiernan, Angelo, Kaufman, & Binder, 2006). Second, PCG was not activated by episodic memory retrieval but rather by stimuli judged as self‐referential, while episodic memory retrieval activated the precuneus (Sajonz et al., 2010). Third, self‐related processes were driven via PCC (Davey et al., 2016). These previous findings suggest that self‐preoccupation is driven via PCG and that the precuneus plays a role in episodic memory retrieval during self‐preoccupation. We speculated that SPS was associated with both vMPFC and PCG. However, SPS was associated with PCG while not associated with vMPFC. The observations were inconsistent with our speculation. A previous study reported that vMPFC and PCC belonged to different subsystems within the default network (Andrews‐Hanna, Smallwood, & Spreng, 2014). The functional difference between vMPFC and PCC may explain the inconsistency between the obtained results and our speculation. Contrary to the previous findings, a recent study reported that vMPFC and PCC belonged to the same default network (Di Plinio & Ebisch, 2018). Therefore, further investigation is needed to determine why a significant association with SPS was not observed in the vMPFC.

The bilateral precentral gyrus, the right postcentral gyrus, and the right supramarginal gyrus showed significant positive associations with SPS, consistent with previous findings that self‐recognition (i.e., face and body) requires activity in precentral regions (Ferri, Frassinetti, Ardizzi, Costantini, & Gallese, 2012; Morita et al., 2008; Sugiura et al., 2006, 2008), postcentral regions (Ferri et al., 2012; Platek et al., 2006; Sugiura et al., 2012), and right supramarginal gyrus (Platek et al., 2006; Sugiura et al., 2006, 2008, 2012). Self‐face is among the most observable representative aspects of the self. Highly self‐preoccupied individuals are expected to focus attention on their faces during rest and feel embarrassment when comparing it to others. This may provide an explanation for the association between SPS and depression (Sakamoto, 1999). On the other hand, we found significant positive associations between the bilateral superior parietal lobules and SPS. A previous neuroimaging study revealed self‐specific (i.e., face and body) activation in the right superior parietal lobule (Sugiura et al., 2006). In addition, the superior parietal lobule has been implicated in episodic memory retrieval (Sajonz et al., 2010). These findings suggest diverse contributions of the superior parietal lobule to self‐preoccupation. It is likely that the superior parietal lobule has a functional role in episodic memory retrieval during self‐preoccupation, particularly in processing information regarding own face and body image. We also found the significant positive associations of SPS with MCG. The cingulate area is divided into three regions, pre‐ and subgenual anterior cingulate cortex, supragenual anterior cingulate cortex (SACC), and PCC (Northoff et al., 2006). The MCG roughly overlaps with SACC and PCC. The SACC has been found to be recruited by both up‐ and downregulation of negative emotion (Ochsner et al., 2004), so the MCG may regulate negative emotions during self‐preoccupation.

The significant negative associations between EPS and rGMV were observed only in the bilateral cerebellum. These results support the second hypothesis and suggest that external‐preoccupation has three attributes, behavioral addiction, sustained attention, and reward system, all of which engage the cerebellum. On the other hand, Internet addicts reportedly showed reduced GMV in regions other than the cerebellum (Weinstein et al., 2017). However, previous findings on GMV reduction in behavioral addiction may reflect both predisposing factors and changes due to ongoing addictive behavior. Given that our study was conducted entirely with healthy subjects, the association between EPS and rGMV in cerebellum may reflect only individual differences in vulnerability to behavioral addiction. A previous neuroimaging study also reported a strong association between the cerebellum and sustained attention (Rosenberg et al., 2016), supporting our speculation that external‐preoccupation is associated with sustained attention. On the other hand, external‐preoccupation represents the tendency to maintain focus for a long time, while capacity for sustained attention is frequently assessed using relatively brief tasks (e.g., 8 min in Esterman, Noonan, Rosenberg, & Degutis, 2013). Therefore, external‐preoccupation and sustained attention may involve distinct neural mechanisms. Nonetheless, our results do suggest that the cerebellum is involved in both short‐ and long‐term attention. According to classical observations, rewards are primarily mediated by the dopaminergic system, including the striatum (Kawagoe, Takikawa, & Hikosaka, 1998; Samejima, Ueda, Doya, & Kimura, 2005) and prefrontal cortex (Hikosaka & Watanabe, 2000, 2004; O'Doherty, Deichmann, Critchley, & Dolan, 2002). If external‐preoccupation is associated with the reward system, significant associations with EPS ought to be observed in canonical reward‐related areas. However, such associations were not observed in the present study. The reasons for this remain uncertain.

RAPM and age were treated as nuisance covariates in the second‐level analysis because these covariates showed significant correlations with SPS. We checked whether our results were indeed influenced by RAPM and age. As a result, the model without RAPM showed similar results to those observed in the model with RAPM, while the model without age showed different results to those observed in the model with age. Therefore, our results were influenced by age but not by RAPM.

If there is an interaction effect between SPS and EPS, it suggests the presence of brain regions related to switching between self‐ and external‐preoccupation or to maintaining focus irrespective of direction of attention. However, no significant interaction effect was observed.

We observed many significant correlations between SPS/EPS and five personality traits. SPS showed a strong positive correlation with neuroticism, the tendency to experience negative emotions and psychological distress in response to stressors (Rosellini & Brown, 2011). The strong positive correlation suggests that self‐preoccupation and neuroticism have similar properties. On the other hand, EPS showed a strong positive correlation with conscientiousness, the level of hardworking (Russell, Woods, & Banks, 2017). Because EPS is associated with burnout, it is reasonable that EPS is positively correlated with conscientiousness.

The present study has two important limitations. First, as emphasized in our previous work (Takeuchi et al., 2017b), our population was restricted to young healthy subjects (18–27 years) with higher levels of education. Further investigation is needed to confirm whether our findings hold true across different age groups and education levels. Second, our hypotheses were mostly based on the previous findings obtained by fMRI. Although there have been a few previous studies on the clear relationship between function and structure (e.g., Papoutsi et al., 2018; Singh et al., 2014), the relationship remains largely unknown. A previous fMRI study revealed that the private self‐consciousness scale, a measure analogous to SPS, correlated with the resting‐state brain activity (Huang et al., 2016). The previous findings suggest not only a relationship between SPS and resting‐state brain activity but also a reciprocal relationship among SPS, rGMV, and resting‐state brain activity. In particular, SPS may be positively associated with regional resting‐state activity of the precuneus and PCG, which showed positive associations between SPS and rGMV. Our future work will investigate the reciprocal relationship among SPS/EPS, rGMV, and regional resting‐state activity.

In conclusion, the present study revealed neural substrates of self‐ and external‐preoccupation at the neurostructural level in a large sample of young subjects. Because self‐preoccupation is the disposition to focus on the self regardless of individual intention, we hypothesized that neural substrates of self‐preoccupation would include the precuneus, vMPFC, and PCC, regions known to be associated with self‐triggered self‐reference. Indeed, significant positive associations with individual differences in self‐preoccupation were observed in the precuneus and PCC, although not in the vMPFC. Our results suggest that vMPFC and PCC play different roles in self‐preoccupation. External‐preoccupation is assumed to have three attributes, behavioral addiction, sustained attention, and reward system, all of which are related to the cerebellum. As expected, significant negative associations with individual differences in external‐preoccupation were observed in the cerebellum. Individual differences in self‐ and external‐preoccupation are implicated in psychopathological states such as depression and burnout. Therefore, investigating neural substrates of self‐ and external‐preoccupation may reveal the neural mechanisms underlying these disorders.
