# Supplementary material for: Neural substrates of self‐ and external‐preoccupation: A voxel‐based morphometry study
Source: Brain Behav. 2019 Apr 19;9(6):e01267. doi: 10.1002/brb3.1267 (PMC6576210; doi:10.1002/brb3.1267)
Supplement: Supplementary file 1 [file BRB3-9-e01267-s001.docx]

**Table S1** Pearson's correlations among demographics of all subjects.

|  | Age | RAPM | TIV | SPS | EPS |
| --- | --- | --- | --- | --- | --- |
| Age | – | 2.0e-03  0.95 | **0.087**  **3.4e-03** | **-0.13**  **1.7e-05** | 2.5e-03  0.93 |
| RAPM |  | – | **0.091**  **2.3e-03** | **-0.080**  **7.1e-03** | -0.016  0.60 |
| TIV |  |  | – | 0.049  0.10 | 9.6e-03  0.75 |
| SPS |  |  |  | – | **0.12**  **3.3e-05** |
| EPS |  |  |  |  | – |

In each cell, upper values indicate correlation values and lower values indicate p values. Bold font indicates correlations that were statistically significant (< 0.05).

RAPM, Raven’s Advanced Progressive Matrices; TIV, Total Intracranial Volume; SPS, Self-Preoccupation Scale; EPS, External-Preoccupation Scale.
