# Supplementary material for: Neural substrates of self‐ and external‐preoccupation: A voxel‐based morphometry study
Source: Brain Behav. 2019 Apr 19;9(6):e01267. doi: 10.1002/brb3.1267 (PMC6576210; doi:10.1002/brb3.1267)
Supplement: Supplementary file 2 [file BRB3-9-e01267-s002.docx]

**Table S2** Pearson's correlations among Preoccupation Scale and five personality traits assessed by the NEO Five-Factor Inventory.

|  | SPS | EPS | N | E | O | A | C |
| --- | --- | --- | --- | --- | --- | --- | --- |
| SPS | – | **0.12**  **3.1e-05** | **0.57**  **1.1e-96** | **-0.18**  **2.5e-09** | **0.16**  **1.6e-07** | **-0.21**  **1.3e-12** | **-0.20**  **1.5e-11** |
| EPS |  | – | 0.049  0.10 | **0.060**  **0.045** | **0.10**  **5.6e-04** | -0.040  0.18 | **0.29**  **1.5e-22** |
| N |  |  | – | **-0.30**  **6.5e-25** | -0.016  0.58 | **-0.27**  **1.4e-20** | **-0.29**  **4.1e-23** |
| E |  |  |  | – | **0.15**  **6.2e-07** | **0.35**  **1.6e-33** | **0.24**  **1.5e-16** |
| O |  |  |  |  | – | **0.11**  **3.5e-04** | -0.018  0.54 |
| A |  |  |  |  |  | – | **0.20**  **3.6e-11** |
| C |  |  |  |  |  |  | – |

In each cell, upper values indicate correlation values and lower values indicate p values. Bold font indicates correlations that were statistically significant (< 0.05).

SPS, Self-Preoccupation Scale; EPS, External-Preoccupation Scale; N, neuroticism; E, extraversion; O, openness; A, agreeableness; C, conscientiousness.
