# Supplementary material for: Neural substrates of self‐ and external‐preoccupation: A voxel‐based morphometry study
Source: Brain Behav. 2019 Apr 19;9(6):e01267. doi: 10.1002/brb3.1267 (PMC6576210; doi:10.1002/brb3.1267)
Supplement: Supplementary file 3 [file BRB3-9-e01267-s003.docx]

**Table S3** Brain areas showing positive associations with Self-Preoccupation Scale in the regression model without RAPM.

|  | Anatomical areas (number of significant voxels of each anatomical area) | x | y | z | TFCE | *P_FWE_* | Cluster  size |
| --- | --- | --- | --- | --- | --- | --- | --- |
| Cluster 1 | R precuneus (1532) | 10.5 | -48 | 63 | 1843.29 | 0.015 | 14527 |
|  | R postcentral gyrus (1214) | 46.5 | -21 | 48 | 2018.74 | 0.011 |  |
|  | R superior parietal lobule (1174) | 33 | -37.5 | 45 | 1905.04 | 0.013 |  |
|  | R precentral gyrus (790) | 45 | 0 | 42 | 1640.91 | 0.023 |  |
|  | R supramarginal gyrus (779) | 49.5 | -25.5 | 49.5 | 1964.21 | 0.011 |  |
|  | R middle cingulate gyrus (729) | 4.5 | -21 | 36 | 1780.2 | 0.017 |  |
|  | R posterior cingulate gyrus (467) | 4.5 | -28.5 | 40.5 | 1772.7 | 0.018 |  |
|  | R middle frontal gyrus (74) | 46.5 | 7.5 | 52.5 | 1546.8 | 0.027 |  |
|  | R postcentral gyrus medial segment (18) | 10.5 | -42 | 61.5 | 1664.84 | 0.022 |  |
|  | R precentral gyrus medial segment (15) | 3 | -27 | 48 | 1598.39 | 0.025 |  |
|  | R cuneus (8) | 1.5 | -75 | 36 | 1406.21 | 0.036 |  |
|  |  |  |  |  |  |  |  |
|  | L middle cingulate gyrus (1052) | 0 | -18 | 37.5 | 1754.75 | 0.018 |  |
|  | L precuneus (778) | 0 | -40.5 | 45 | 1678.92 | 0.022 |  |
|  | L posterior cingulate gyrus (627) | 0 | -27 | 45 | 1737.45 | 0.02 |  |
|  | L superior parietal lobule (345) | -15 | -73.5 | 48 | 1398.66 | 0.037 |  |
|  | L precentral gyrus medial segment (177) | -3 | -24 | 46.5 | 1692.83 | 0.021 |  |
|  | L angular gyrus (104) | -27 | -66 | 34.5 | 1388.96 | 0.038 |  |
|  | L precentral gyrus (3) | -24 | -13.5 | 52.5 | 1295.95 | 0.048 |  |
|  | L cuneus (1) | 0 | -76.5 | 36 | 1328.59 | 0.045 |  |
|  | L supplementary motor cortex (1) | -4.5 | -18 | 46.5 | 1295.95 | 0.048 |  |
|  |  |  |  |  |  |  |  |
|  | * R cerebral white matter (2684) | 45 | -22.5 | 49.5 | 2013.63 | 0.011 |  |
|  | * L cerebral white matter (1239) | -9 | -33 | 37.5 | 1667.47 | 0.022 |  |
|  | * Unknown (716) | 51 | -24 | 49.5 | 1955.25 | 0.012 |  |
| Cluster 2 | * R cerebral white matter (18) | 13.5 | -43.5 | 27 | 1296.43 | 0.048 | 18 |
| Cluster 3 | R superior parietal lobule (5) | 42 | -52.5 | 57 | 1284.48 | 0.049 | 12 |
|  | R angular gyrus (2) | 42 | -51 | 55.5 | 1282.19 | 0.049 |  |
|  |  |  |  |  |  |  |  |
|  | * Unknown (5) | 40.5 | -52.5 | 57 | 1284.06 | 0.049 |  |

Labeling of brain areas is conducted using custom Matlab scripts and labels_Neuromorphometrics.nii in SPM12. The coordinates of the peak voxel of each brain area are shown as x, y, and z. Asterisks represent white matter and areas that could not be labeled. The TFCE magnitude and corrected p-value (FWE) for each peak voxel were shown. Cluster size represents the number of voxels which each cluster includes. R: right; L: left.
