# Supplementary material for: Neural substrates of self‐ and external‐preoccupation: A voxel‐based morphometry study
Source: Brain Behav. 2019 Apr 19;9(6):e01267. doi: 10.1002/brb3.1267 (PMC6576210; doi:10.1002/brb3.1267)
Supplement: Supplementary file 4 [file BRB3-9-e01267-s004.docx]

**Table S4** Brain areas showing negative associations with External-Preoccupation Scale in the regression model without RAPM.

|  | Anatomical areas (number of significant voxels of each anatomical area) | x | y | z | TFCE | *P_FWE_* | Cluster size |
| --- | --- | --- | --- | --- | --- | --- | --- |
| Cluster 1 | L cerebellum exterior (3082) | -33 | -66 | -45 | 1699.67 | 0.018 | 4458 |
|  |  |  |  |  |  |  |  |
|  | * L cerebellum white matter (971) | -27 | -55.5 | -40.5 | 1724.31 | 0.017 |  |
|  | * Unknown (405) | -22.5 | -55.5 | -63 | 1481.02 | 0.028 |  |
| Cluster 2 | R cerebellum exterior (5820) | 18 | -79.5 | -36 | 2219.54 | 0.006 | 7457 |
|  |  |  |  |  |  |  |  |
|  | Cerebellar vermal lobules viii−x (91) | 7.5 | -67.5 | -37.5 | 1751.83 | 0.017 |  |
|  | Cerebellar vermal lobules vi−vii (2) | 6 | -66 | -31.5 | 1318.9 | 0.043 |  |
|  |  |  |  |  |  |  |  |
|  | * R cerebellum white matter (1022) | 16.5 | -75 | -37.5 | 2181.14 | 0.007 |  |
|  | * Unknown (522) | 18 | -91.5 | -27 | 1887.17 | 0.012 |  |

Labeling of brain areas is conducted using custom Matlab scripts and labels_Neuromorphometrics.nii in SPM12. The coordinates of the peak voxel of each brain area are shown as x, y, and z. Asterisks represent white matter and areas that could not be labeled. The TFCE magnitude and corrected p-value (FWE) for each peak voxel were shown. Cluster size represents the number of voxels which each cluster includes. R: right; L: left.
