# Supplementary material for: Neural substrates of self‐ and external‐preoccupation: A voxel‐based morphometry study
Source: Brain Behav. 2019 Apr 19;9(6):e01267. doi: 10.1002/brb3.1267 (PMC6576210; doi:10.1002/brb3.1267)
Supplement: Supplementary file 5 [file BRB3-9-e01267-s005.docx]

**Table S5** Brain areas showing positive associations with Self-Preoccupation Scale in the regression model without age.

|  | Anatomical areas (number of significant voxels of each anatomical area) | x | y | z | TFCE | *P_FWE_* | Cluster size |
| --- | --- | --- | --- | --- | --- | --- | --- |
| Cluster 1 | R middle frontal gyrus (4315) | 46.5 | 7.5 | 52.5 | 2409.82 | 0.006 | 121203 |
|  | R precuneus (3256) | 3 | -39 | 45 | 3116.67 | 0.001 |  |
|  | R postcentral gyrus (2219) | 46.5 | -21 | 48 | 3051.97 | 0.001 |  |
|  | R superior parietal lobule (2123) | 37.5 | -36 | 45 | 2922.41 | 0.002 |  |
|  | R superior frontal gyrus (1855) | 27 | 61.5 | 1.5 | 2342.61 | 0.006 |  |
|  | R precentral gyrus (1820) | 45 | 0 | 42 | 2538.02 | 0.004 |  |
|  | R middle cingulate gyrus (1523) | 4.5 | -27 | 40.5 | 3158.09 | 0.001 |  |
|  | R supramarginal gyrus (1463) | 49.5 | -27 | 52.5 | 2994.78 | 0.002 |  |
|  | R putamen (1458) | 22.5 | 18 | 0 | 1884.18 | 0.015 |  |
|  | R angular gyrus (1352) | 42 | -51 | 55.5 | 2085.47 | 0.01 |  |
|  | R posterior cingulate gyrus (1268) | 4.5 | -28.5 | 40.5 | 3158.09 | 0.001 |  |
|  | R frontal pole (1021) | 27 | 64.5 | 1.5 | 2327.29 | 0.006 |  |
|  | R triangular part of the inferior frontal gyrus (999) | 51 | 45 | -3 | 2288.08 | 0.006 |  |
|  | R temporal pole (837) | 48 | 7.5 | -34.5 | 1789.15 | 0.018 |  |
|  | R medial orbital gyrus (834) | 12 | 45 | -24 | 2186.37 | 0.008 |  |
|  | R middle temporal gyrus (805) | 51 | 4.5 | -34.5 | 1716.3 | 0.019 |  |
|  | R lateral orbital gyrus (620) | 48 | 46.5 | -10.5 | 2125.9 | 0.009 |  |
|  | R anterior insula (617) | 42 | 19.5 | -1.5 | 2209.86 | 0.007 |  |
|  | R superior frontal gyrus medial segment (616) | 1.5 | 58.5 | 1.5 | 1872.31 | 0.016 |  |
|  | R inferior temporal gyrus (582) | 48 | -7.5 | -34.5 | 1682.41 | 0.02 |  |
|  | R lingual gyrus (544) | 3 | -58.5 | 9 | 1957.54 | 0.013 |  |
|  | R orbital part of the inferior frontal gyrus (469) | 49.5 | 45 | -9 | 2238.51 | 0.007 |  |
|  | R gyrus rectus (427) | 1.5 | 30 | -24 | 2175.61 | 0.008 |  |
|  | R frontal operculum (396) | 51 | 21 | -3 | 2276.62 | 0.007 |  |
|  | R precentral gyrus medial segment (393) | 3 | -27 | 48 | 2957.28 | 0.002 |  |
|  | R lateral ventricle (365) | 7.5 | -16.5 | 24 | 2141.01 | 0.009 |  |
|  | R supplementary motor cortex (355) | 7.5 | -16.5 | 46.5 | 2041.69 | 0.011 |  |
|  | R caudate (351) | 12 | 21 | -7.5 | 1878.51 | 0.015 |  |
|  | R medial frontal cortex (346) | 0 | 28.5 | -24 | 2177.21 | 0.008 |  |
|  | R entorhinal area (341) | 16.5 | 0 | -19.5 | 1994.82 | 0.012 |  |
|  | R amygdala (328) | 15 | -1.5 | -19.5 | 1974.63 | 0.012 |  |
|  | R posterior orbital gyrus (317) | 22.5 | 21 | -24 | 1969.49 | 0.013 |  |
|  | R pallidum (247) | 18 | 7.5 | 0 | 1804.36 | 0.018 |  |
|  | R anterior orbital gyrus (239) | 28.5 | 58.5 | -6 | 2284.33 | 0.007 |  |
|  | R superior temporal gyrus (145) | 60 | -48 | 13.5 | 1440.9 | 0.036 |  |
|  | R hippocampus (140) | 18 | -4.5 | -25.5 | 1804.36 | 0.018 |  |
|  | R opercular part of the inferior frontal gyrus (132) | 52.5 | 21 | -1.5 | 2268.79 | 0.007 |  |
|  | R anterior cingulate gyrus (123) | 1.5 | 24 | 22.5 | 1890.79 | 0.015 |  |
|  | R postcentral gyrus medial segment (108) | 10.5 | -42 | 63 | 2809.89 | 0.002 |  |
|  | R posterior insula (103) | 39 | 6 | -13.5 | 1825.98 | 0.017 |  |
|  | R cuneus (86) | 1.5 | -75 | 36 | 2554.84 | 0.004 |  |
|  | R central operculum (82) | 63 | -15 | 18 | 1890.79 | 0.015 |  |
|  | R parietal operculum (81) | 61.5 | -25.5 | 21 | 1730.57 | 0.019 |  |
|  | R basal forebrain (69) | 21 | 3 | -15 | 1936.09 | 0.014 |  |
|  | R planum polare (61) | 43.5 | 6 | -16.5 | 1639.86 | 0.021 |  |
|  | R accumbens area (56) | 13.5 | 19.5 | -9 | 1873.81 | 0.015 |  |
|  | R parahippocampal gyrus (51) | 12 | -4.5 | -21 | 1869.46 | 0.016 |  |
|  | R calcarine cortex (34) | 3 | -63 | 12 | 1890.79 | 0.015 |  |
|  | R planum temporale (22) | 63 | -25.5 | 19.5 | 1675.9 | 0.02 |  |
|  | R superior occipital gyrus (8) | 30 | -73.5 | 43.5 | 1625.49 | 0.022 |  |
|  | R ventral dc (7) | 10.5 | -1.5 | -18 | 1851.04 | 0.016 |  |
|  | R middle occipital gyrus (5) | 33 | -75 | 45 | 1569.28 | 0.026 |  |
|  | R subcallosal area (4) | 0 | 18 | -21 | 1447.35 | 0.035 |  |
|  | R cerebellum exterior (2) | 13.5 | -48 | -9 | 1306.23 | 0.05 |  |
|  | R inf lat vent (1) | 30 | -6 | -27 | 1306.23 | 0.05 |  |
|  |  |  |  |  |  |  |  |
|  | L middle frontal gyrus (3074) | -31.5 | 0 | 54 | 1882.08 | 0.015 |  |
|  | L precuneus (2611) | 0 | -40.5 | 45 | 3004 | 0.001 |  |
|  | L angular gyrus (2216) | -27 | -66 | 34.5 | 2292.15 | 0.006 |  |
|  | L middle temporal gyrus (2116) | -58.5 | -54 | 12 | 2228.62 | 0.007 |  |
|  | L middle cingulate gyrus (1942) | 0 | -25.5 | 45 | 3130.71 | 0.001 |  |
|  | L middle occipital gyrus (1630) | -27 | -78 | 37.5 | 2241.04 | 0.007 |  |
|  | L postcentral gyrus (1624) | -19.5 | -36 | 60 | 1940.83 | 0.013 |  |
|  | L putamen (1547) | -30 | 3 | 4.5 | 1784.59 | 0.018 |  |
|  | L precentral gyrus (1330) | -25.5 | -13.5 | 52.5 | 2241.04 | 0.007 |  |
|  | L superior parietal lobule (1274) | -15 | -73.5 | 46.5 | 2431.08 | 0.006 |  |
|  | L posterior cingulate gyrus (1261) | 0 | -27 | 45 | 3116.67 | 0.001 |  |
|  | L superior temporal gyrus (1138) | -58.5 | -52.5 | 12 | 2230.74 | 0.007 |  |
|  | L superior frontal gyrus (1095) | -22.5 | -12 | 51 | 2141.01 | 0.009 |  |
|  | L fusiform gyrus (1062) | -46.5 | -55.5 | -24 | 1621.75 | 0.023 |  |
|  | L supramarginal gyrus (974) | -60 | -58.5 | 27 | 2210.47 | 0.007 |  |
|  | L triangular part of the inferior frontal gyrus (956) | -43.5 | 33 | 13.5 | 1701.8 | 0.02 |  |
|  | L lingual gyrus (935) | -9 | -91.5 | -9 | 1537.19 | 0.028 |  |
|  | L precentral gyrus medial segment (907) | -1.5 | -31.5 | 49.5 | 3084.09 | 0.001 |  |
|  | L anterior insula (889) | -34.5 | 4.5 | 3 | 1782.55 | 0.018 |  |
|  | L medial orbital gyrus (857) | -7.5 | 55.5 | -22.5 | 2252.64 | 0.007 |  |
|  | L cerebellum exterior (819) | -49.5 | -54 | -27 | 1619.51 | 0.023 |  |
|  | L inferior temporal gyrus (815) | -51 | -55.5 | -25.5 | 1623.88 | 0.022 |  |
|  | L anterior cingulate gyrus (795) | -1.5 | 24 | 24 | 2388.13 | 0.006 |  |
|  | L gyrus rectus (782) | -4.5 | 58.5 | -21 | 2259.15 | 0.007 |  |
|  | L opercular part of the inferior frontal gyrus (729) | -42 | 10.5 | 28.5 | 1681.71 | 0.02 |  |
|  | L inferior occipital gyrus (689) | -37.5 | -91.5 | 6 | 2179.69 | 0.008 |  |
|  | L medial frontal cortex (454) | 0 | 30 | -24 | 2178.09 | 0.008 |  |
|  | L frontal pole (449) | -4.5 | 61.5 | -22.5 | 2248.31 | 0.007 |  |
|  | L superior frontal gyrus medial segment (419) | -1.5 | 57 | 0 | 1822.77 | 0.017 |  |
|  | L entorhinal area (375) | -19.5 | 1.5 | -21 | 1632.7 | 0.022 |  |
|  | L frontal operculum (364) | -39 | 9 | 3 | 1678.39 | 0.02 |  |
|  | L postcentral gyrus medial segment (335) | -3 | -39 | 54 | 2429.43 | 0.006 |  |
|  | L occipital fusiform gyrus (305) | -30 | -88.5 | -16.5 | 1652.53 | 0.021 |  |
|  | L pallidum (267) | -24 | -3 | 0 | 1689.62 | 0.02 |  |
|  | L occipital pole (265) | -28.5 | -96 | 7.5 | 2084.81 | 0.01 |  |
|  | L supplementary motor cortex (240) | -4.5 | -18 | 46.5 | 2627.95 | 0.003 |  |
|  | L amygdala (213) | -21 | -1.5 | -22.5 | 1579.22 | 0.025 |  |
|  | L parahippocampal gyrus (212) | -24 | -15 | -31.5 | 1537.33 | 0.028 |  |
|  | L superior occipital gyrus (170) | -27 | -94.5 | 9 | 2074.42 | 0.01 |  |
|  | L calcarine cortex (165) | -9 | -91.5 | -7.5 | 1535.04 | 0.028 |  |
|  | L anterior orbital gyrus (129) | -25.5 | 61.5 | -6 | 1725.23 | 0.019 |  |
|  | L posterior orbital gyrus (108) | -21 | 18 | -21 | 1889.43 | 0.015 |  |
|  | L hippocampus (86) | -24 | -10.5 | -28.5 | 1535.97 | 0.028 |  |
|  | L central operculum (83) | -39 | 6 | 6 | 1678.39 | 0.02 |  |
|  | L posterior insula (77) | -37.5 | -4.5 | 0 | 1611.86 | 0.024 |  |
|  | L caudate (74) | -13.5 | 13.5 | -4.5 | 1597.19 | 0.024 |  |
|  | L basal forebrain (47) | -21 | 4.5 | -18 | 1547.85 | 0.027 |  |
|  | L orbital part of the inferior frontal gyrus (46) | -48 | 27 | -7.5 | 1551.39 | 0.027 |  |
|  | L accumbens area (31) | -15 | 16.5 | -9 | 1567.38 | 0.026 |  |
|  | L planum temporale (31) | -61.5 | -42 | 21 | 1569.28 | 0.026 |  |
|  | L temporal pole (21) | -21 | 4.5 | -28.5 | 1541.14 | 0.028 |  |
|  | L cuneus (16) | 0 | -76.5 | 36 | 2241.04 | 0.007 |  |
|  | L subcallosal area (16) | -1.5 | 19.5 | -21 | 1716.91 | 0.019 |  |
|  | L lateral ventricle (1) | -19.5 | -45 | 6 | 1306.23 | 0.05 |  |
|  | L vessel (1) | -27 | -1.5 | -9 | 1306.23 | 0.05 |  |
|  | L parietal operculum (1) | -60 | -40.5 | 24 | 1306.23 | 0.05 |  |
|  |  |  |  |  |  |  |  |
|  | * R cerebral white matter (15016) | 7.5 | -21 | 34.5 | 3153.06 | 0.001 |  |
|  | * L cerebral white matter (15593) | -7.5 | -31.5 | 39 | 3026.6 | 0.001 |  |
|  | * Unknown (15505) | 0 | -34.5 | 49.5 | 3143.93 | 0.001 |  |
| Cluster 2 | R superior frontal gyrus (1) | 27 | 7.5 | 60 | 1306.23 | 0.05 | 1 |

Labeling of brain areas is conducted using custom Matlab scripts and labels_Neuromorphometrics.nii in SPM12. The coordinates of the peak voxel of each brain area are shown as x, y, and z. Asterisks represent white matter and areas that could not be labeled. The TFCE magnitude and corrected p-value (FWE) for each peak voxel were shown. Cluster size represents the number of voxels which each cluster includes. R: right; L: left.
